# Reductive Augmentation of the Breast

**DOI:** 10.1007/s00266-017-1010-0

**Published:** 2017-11-09

**Authors:** Paul E. Chasan

**Affiliations:** Ranch and Coast Plastic Surgery, 1431 Camino del Mar, Del Mar, CA 92014 USA

**Keywords:** Reductive augmentation, Breast reduction, Breast augmentation, Breast lift, Mastopexy, Round breast

## Abstract

**Aim:**

Although breast reduction surgery plays an invaluable role in the correction of macromastia, it almost always results in a breast lacking in upper pole fullness and/or roundness. We present a technique of breast reduction combined with augmentation termed “reductive augmentation” to solve this problem. The technique is also extremely useful for correcting breast asymmetry, as well as revising significant pseudoptosis in the patient who has previously undergone breast augmentation with or without mastopexy.

**Methods:**

An evolution of techniques has been used to create a breast with more upper pole fullness and anterior projection in those patients desiring a more round, higher-profile appearance. Reductive augmentation is a one-stage procedure in which a breast augmentation is immediately followed by a modified superomedial pedicle breast reduction. Often, the excision of breast tissue is greater than would normally be performed with breast reduction alone.

**Results:**

Thirty-five patients underwent reductive augmentation, of which 12 were primary surgeries and 23 were revisions. There was an average tissue removal of 255 and 227 g, respectively, per breast for the primary and revision groups. Six of the reductive augmentations were performed for gross asymmetry. Fourteen patients had a previous mastopexy, and 3 patients had a previous breast reduction. The average follow-up was 26 months.

**Conclusions:**

Reductive augmentation is an effective one-stage method for achieving a more round-appearing breast with upper pole fullness both in primary breast reduction candidates and in revisionary breast surgery. This technique can also be applied to those patients with significant asymmetry.

**Level of Evidence IV:**

This journal requires that authors assign a level of evidence to each article. For a full description of these Evidence-Based Medicine ratings, please refer to the Table of Contents or the online Instructions to Authors www.springer.com/00266.

## Introduction

**Fig. 1:**
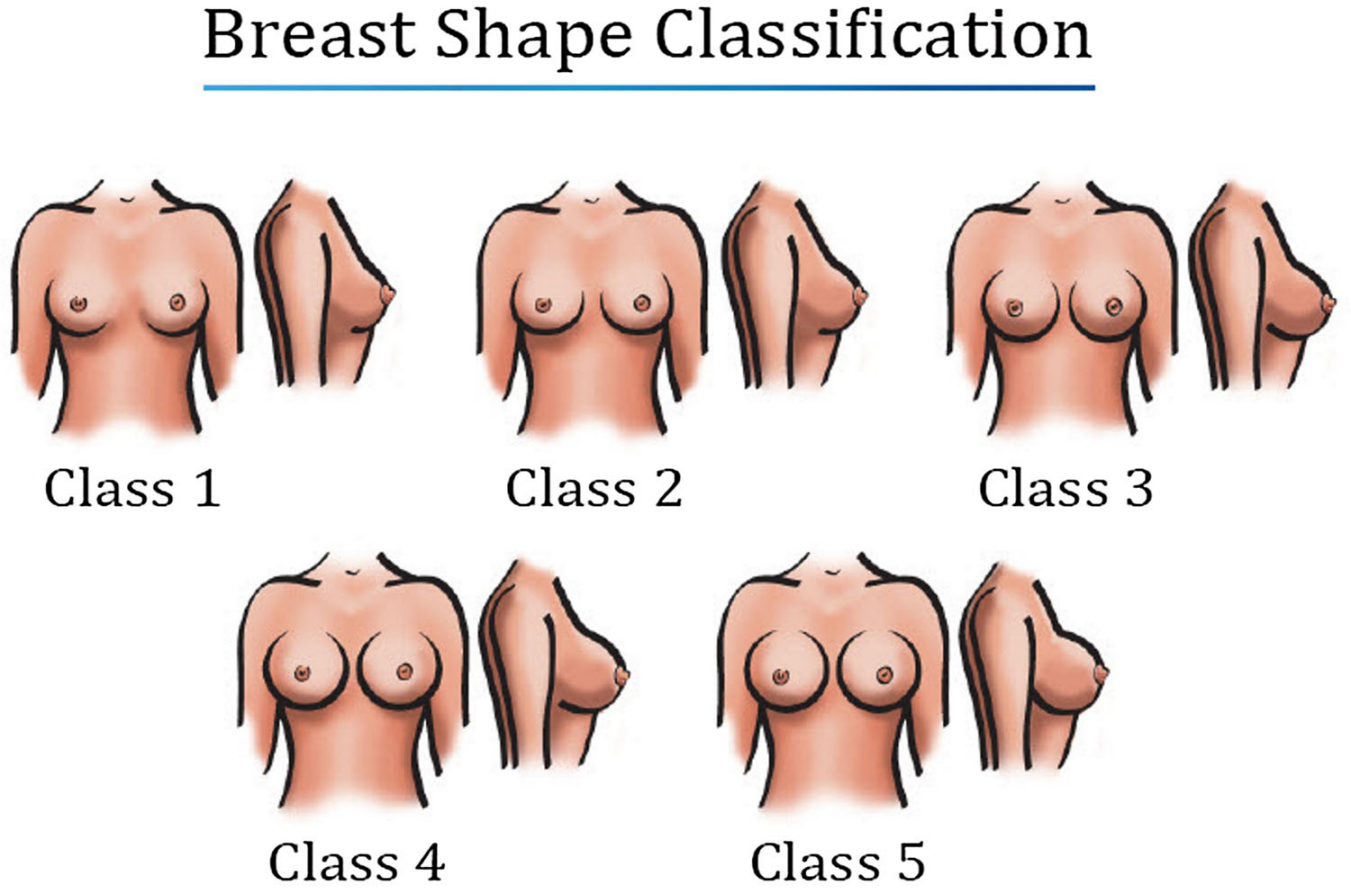
Breast shape classification: Class 1—appears natural without implant contour, Class 2—appears natural with slight implant contour, Class 3—intermediate in roundness of upper pole, Class 4—round appearance of upper pole, Class 5—maximum roundness of upper pole

**Fig. 2:**
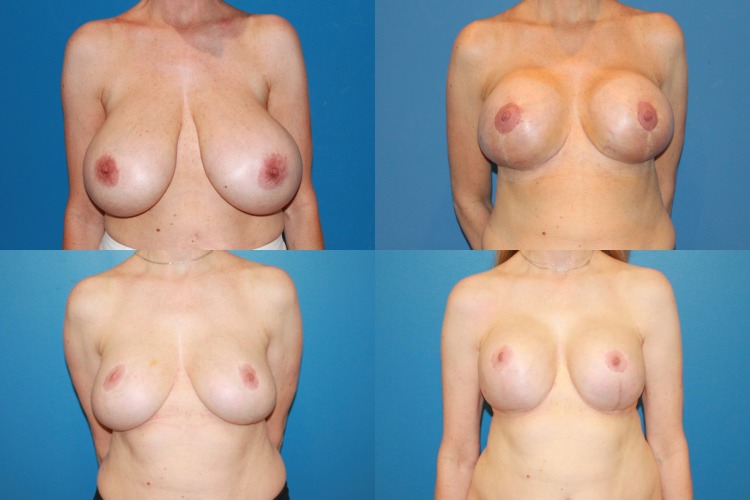
A 54-year-old female G2P2 with 36 G cup breasts who wanted to have large round breasts after breast reduction (sternal notch-to-nipple distance 30 cm). **a** Preoperative, **b** 1 month after breast reduction removing 455/585 g, **c** 6 months postoperatively—satisfactory result, but patient unhappy as she had little upper pole fullness, **d** 8 months after reductive augmentation (Allergan Style 20, 280 cc placed with excision of 275 g from the right breast and 245 g from the left breast)

Breast reduction is an important tool in the plastic surgeon’s armamentarium. There are few plastic surgery procedures that result in as a high level of patient satisfaction. Multiple generations of the technique have resulted in better shape, less scars, and more predictable results [1–6]. However, even with the most proficient and technically advanced breast reductions, there continue to be limitations with respect to the shape of the breast. With time, there is almost always a lack of superior pole fullness [7]. Although many patients are satisfied with the results from the current art of breast reduction, there are a number who request a more “perky” or “round” result and/or desire a breast with a rounder shape and upper pole fullness, a class 3-5/5 based on a breast shape classification system (Figs. 1) [8]. Reductive augmentation is a surgical procedure that has been developed to achieve this type of result (Fig. 2).

Reductive augmentation is a one-stage, superomedial-based pedicle breast reduction in conjunction with subpectoral breast augmentation with silicone implants. The concept of removing tissue only to have it replaced by an implant may seem somewhat counterintuitive; however, by removing a volume of tissue that exceeds the implanted volume, an overall smaller breast with more superior pole fullness and more anterior projection is fashioned. This rounder, fuller result is more aesthetically appealing to a specific but substantial subset of our breast reduction population.

Though the procedure was initially developed to provide a superior cosmetic result in cases of macromastia, reductive augmentation has proven to be a versatile and effective technique with other useful applications: patients with redundant lower pole tissue and pseudoptosis after previous breast augmentation with or without mastopexy. In such cases, the reductive portion of the procedure is performed in conjunction with an implant exchange to a higher-profile implant, a lateral or inferolateral capsulorrhaphy, and mirror-image capsulotomy [9, 10]. In all cases of subglandular position of the implant, a site change was performed to the subpectoral position. Also, reductive augmentation can be used to correct grossly asymmetric breasts, either occurring primarily or following previous augmentation. In this situation, tissue is removed disproportionately from the larger breast such that implants of the same size can be placed bilaterally with a symmetric result.

The current version of reductive augmentation has had several revisions since first starting this procedure in 2003 and now is hopefully in its final form. The technical aspects of reductive augmentation are outlined, and the data presented from patients over the last 4 years who underwent the procedure.

## Patients Selection and Methods

Initially, reductive augmentation was offered to patients who desired smaller breasts with more superior pole fullness and anterior projection than could be achieved with standard breast reduction techniques. The selection criteria were expanded to include patients with excess inferior pole breast tissue (pseudoptosis) following prior breast augmentation with or without mastopexy, as well as patients with gross breast asymmetry. Although the procedure had been done for many years, it had undergone a variety of modifications. The start of the study represents the technique in its most recent form.

## Surgical Technique

### Preoperative Marking

Prior to surgery and with the patient upright, the inframammary creases and breast meridians are marked. The position of the new nipple-areolar complex is approximated and marked by a blotting technique. At this point, the breasts are compared in size and the amount of tissue removal that will be necessary is estimated. When asymmetry is observed, the difference between the amounts of tissue removed from each breast will be guided by an estimation of the initial size discrepancy, with a goal of establishing symmetry following placement of same-sized implants bilaterally. Next, the areola-to-inframammary crease distance is measured. This distance gives a prediction as to the width and length of the horizontal excision in the inframammary crease. Lastly, the distance from sternal notch to inframammary crease (IMC) is measured to estimate the elevation of the IMC.

### Subpectoral Pocket Dissection and Sizer Implant Placement

The procedure is performed under general anesthesia with the patient positioned in akimbo (hands taped to the anterior iliac spine and elbows padded at the edge of the table). A gel pad is placed under the torso, so the patient will not slide down the bed when placed in a sitting position. A standard subpectoral breast augmentation is performed through an infra-areolar vertical incision. A 2-cm transverse incision is made in the pectoralis major muscle and dilated via retraction, and a dual-plane/partial submuscular pocket is developed. Lateral dissection is minimized to keep the implant in a more medial location. Based on the patient’s desires and anatomy, either a style 45/SRX or style 20/SRF sizer (Allergan, Inc., Irvine, CA, USA) is placed, and the posterior breast tissue and pectoralis major muscle are closed temporarily with a few figure-of-eight sutures. If the patient has had a previous breast augmentation, a sizer is placed after performing a lateral or inferolateral capsulorrhaphy and mirror-image capsulotomy [10, 11]. In patients who have a subglandular implant, a site change to a subpectoral location is performed if indicated. The indications for site change are recurrent capsular contracture, excessive wrinkling, and/or implant slide down.

### Tailor-Tack Mastopexy

With the patient in an upright seated position, a tailor-tack mastopexy [12, 13] is then performed with staples. The identical procedure is performed on the contralateral side to insure symmetry. The incisions are appropriately marked, and the staples are removed.

### Tissue Resection

A 38-mm cookie cutter is then used to mark the areola, and the mastopexy and areolar incisions are made. An 8-cm-wide superomedial pedicle is de-epithelialized as well as a 5-mm cuff around the new areolar opening. The intervening superior and lateral periareolar tissue is excised down to pectoral major muscular fascia along with the lateral and inferior pole breast tissue. The remaining superior, lateral, and limited medial flaps are then elevated off the periprosthetic remaining tissue or, in cases of previous breast augmentation, off the pericapsular tissue. In larger excisions, it is important to elevate the flaps extensively to prevent a flattening of the inferior pole of the breast. A determination is then made if further resection is warranted based on the thickness of the flaps, final desired size, and roundness based on the patient’s anatomy and preoperative aspirations. If significant bulk remains in the medial and lateral aspects of larger breasts, additional tissue removal is performed to leave 2.0-cm-thick flaps. It is important to try to match the thickness of the medial and lateral flaps. A common mistake is to leave too much thickness on the medial or thin the lateral flap too aggressively giving an unbalanced appearance.

### Secondary Mastopexy, External Capsulorrhaphy, and Final Implant Placement

The tissue resection significantly alters the shape of the breast, and the previous mastopexy markings result in relative skin laxity. This requires further refinement in the mastopexy markings to achieve the best shape. Additionally, the resulting circumareolar opening becomes too large. In the upright/sitting up position, a “secondary” tailor-tack mastopexy is then performed to adjust and finalize the original mastopexy markings, improve symmetry, and reduce the size of the areolar opening. Once satisfied with the markings, the final size and style of the implant are determined, the pocket irrigated with triple antibiotic solution followed by Betadine, and the appropriate implant placed. The pectoralis major and breast tissue surrounding the implant or capsular tissue (revisionary implant cases) are closed vertically with 2-0 Vicryl (polyglactin 910, Ethicon, Inc.) sutures. Following this, several horizontal mattress sutures are placed to imbricate the vertical closure and further tighten the inferior pole of the pocket, hence the term “external capsulorrhaphy.” A 7-mm Jackson–Pratt drain is then placed. In most cases, there is a dramatic elevation of the inframammary crease, and a transverse crescent-shaped excision is necessary to remove redundant skin at the inferior aspect of the breast.

### Closure and Insetting the Areola

After the modified mastopexy incisions are made and the intervening skin de-epithelialized, the pillars of breast tissue from the remaining medial and lateral flaps are closed with interrupted 2-0 Vicryl sutures, taking tension off the vertical closure. The vertical incision is then closed in a standard fashion with interrupted dermal and running subcuticular 4-0 Monocryl (poliglecaprone 25, Ethicon, Inc.) sutures. The periareolar tissues are purse-stringed [12] to an approximate areolar diameter of 30 mm with a dermal CV3 Gore-tex (expanded polytetrafluoroethylene [ePTFE], WL Gore & Associates) suture. A 36-mm cookie cutter is used again to mark the final placement of the areola in an upright position, and the periareolar tissue is de-epithelialized. Finally, the areola is inset with four external interrupted 5-0 nylon sutures followed by a running subcuticular 4-0 PDS suture.

It should be noted that after each maneuver on each side, the patient is sat up. The average number of times the patient is sat up during surgery is 12.

### Postoperative Management

Postoperatively, the patient is placed in a standard compressive breast dressing, which is changed to an athletic brassiere on the first postoperative day. The drain is removed on postoperative day 2–4 or until the drainage is less than 30 cc per day. The remainder of the postoperative care is similar to a standard breast augmentation and mastopexy.

## Results

**Fig. 3:**
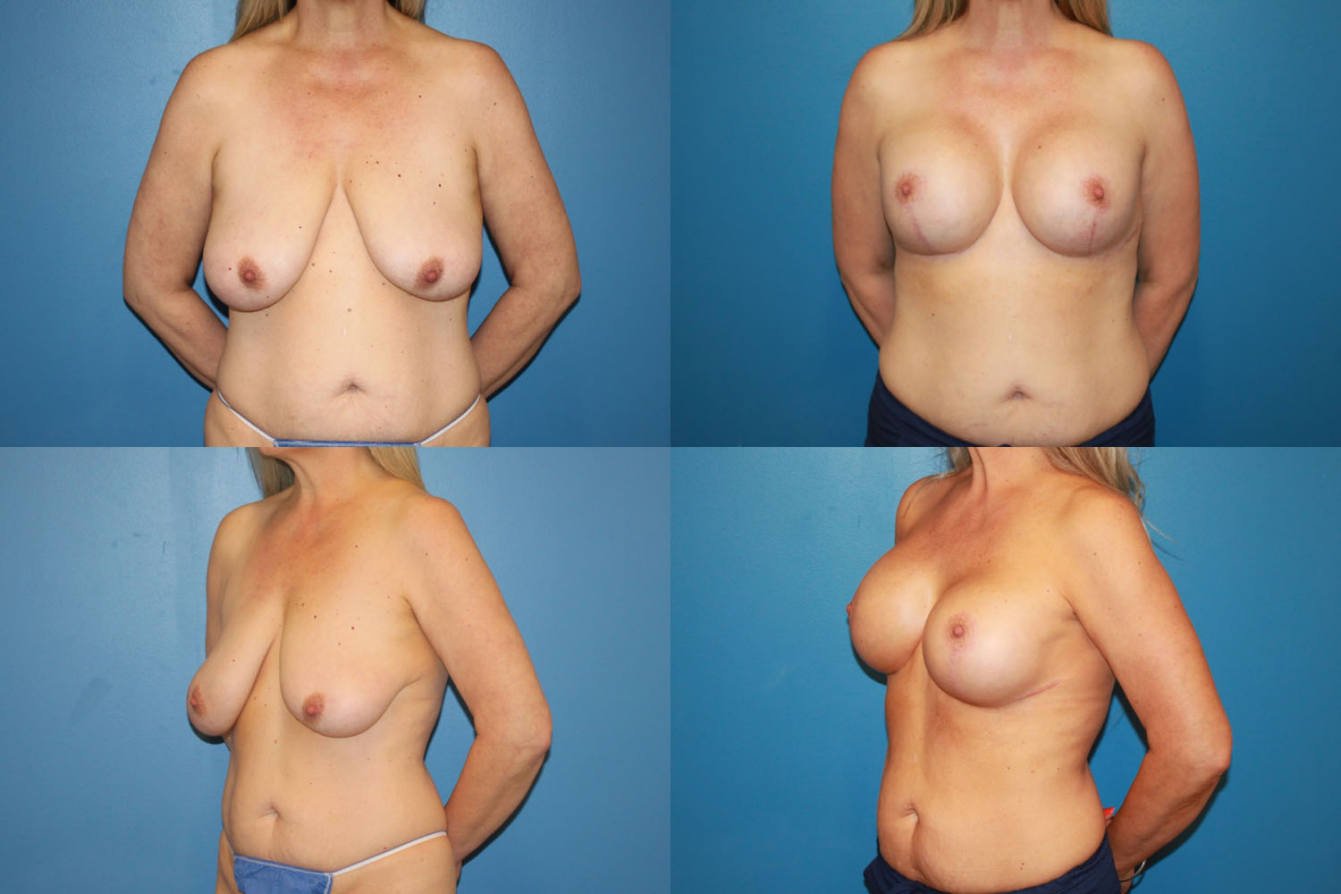
A 52-year-old female G4P3 with 36 DD breasts and grade 3 ptosis (sternal notch-to-nipple distance 29 cm) who desired to be a 36 small D cup with 4/5 in definition. Reductive augmentation was performed removing 335 g bilaterally and placing Allergan SRX 470 cc ultra-high-profile silicone implants bilaterally. Seven months postoperatively

**Fig. 4:**
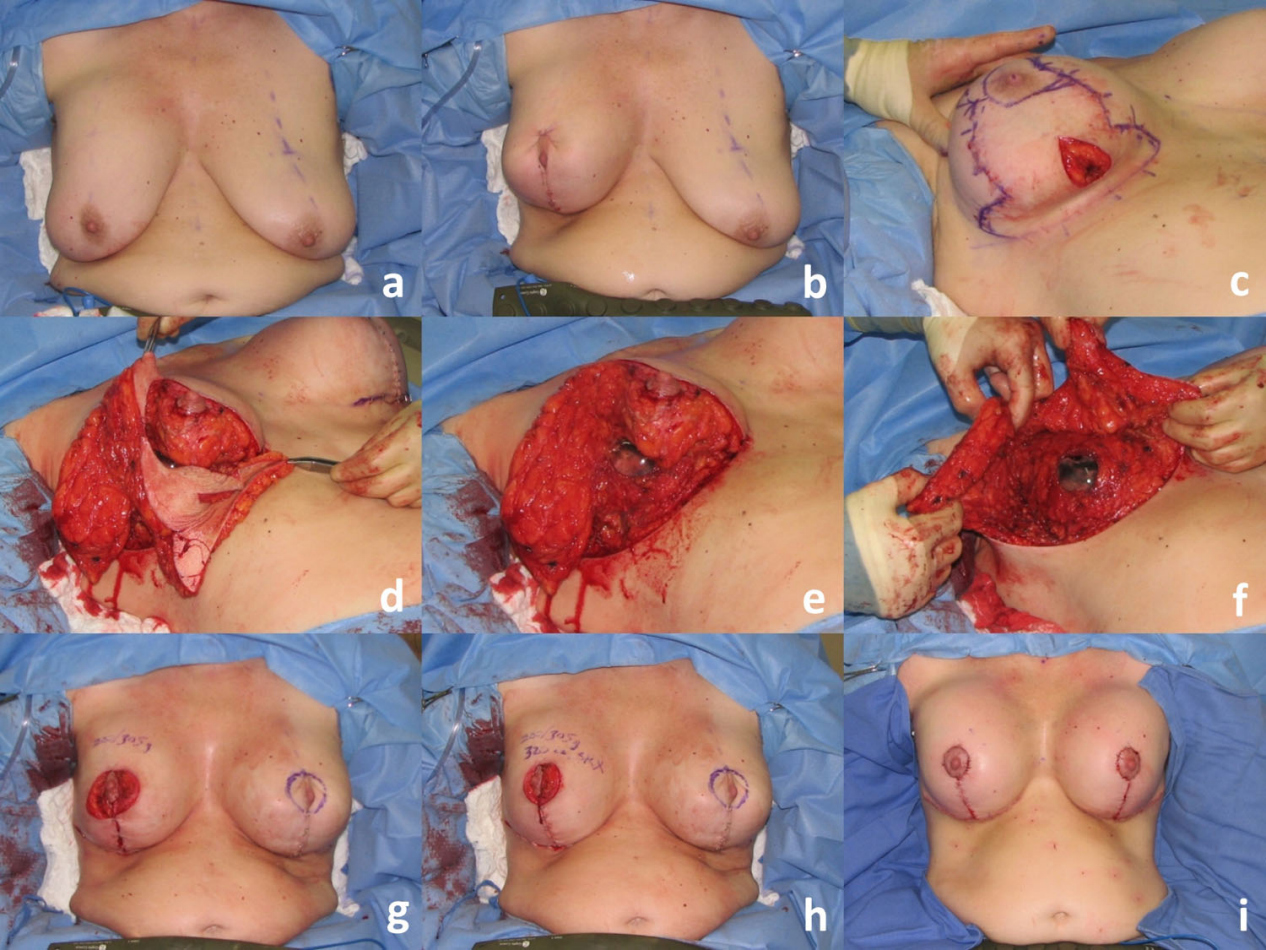
Operative sequence of patient in Fig. 2: **a** placement of right breast implant sizer (320 cc Style 45) via infra-areolar vertical incision, **b** tailor-tack mastopexy with staples in sitting position, **c** markings made and staples removed, note superomedial pedicle marked, **d** initial excision (220 g) before removal, **e** after removal of specimen—noted thickness of flaps, **f** lateral and medial flaps after thinning/excision (additional 115 g), **g** re-stapling, **h** after secondary mastopexy, **i** final closure after placing implant (SRX 470 cc) in sitting position

**Fig. 5:**
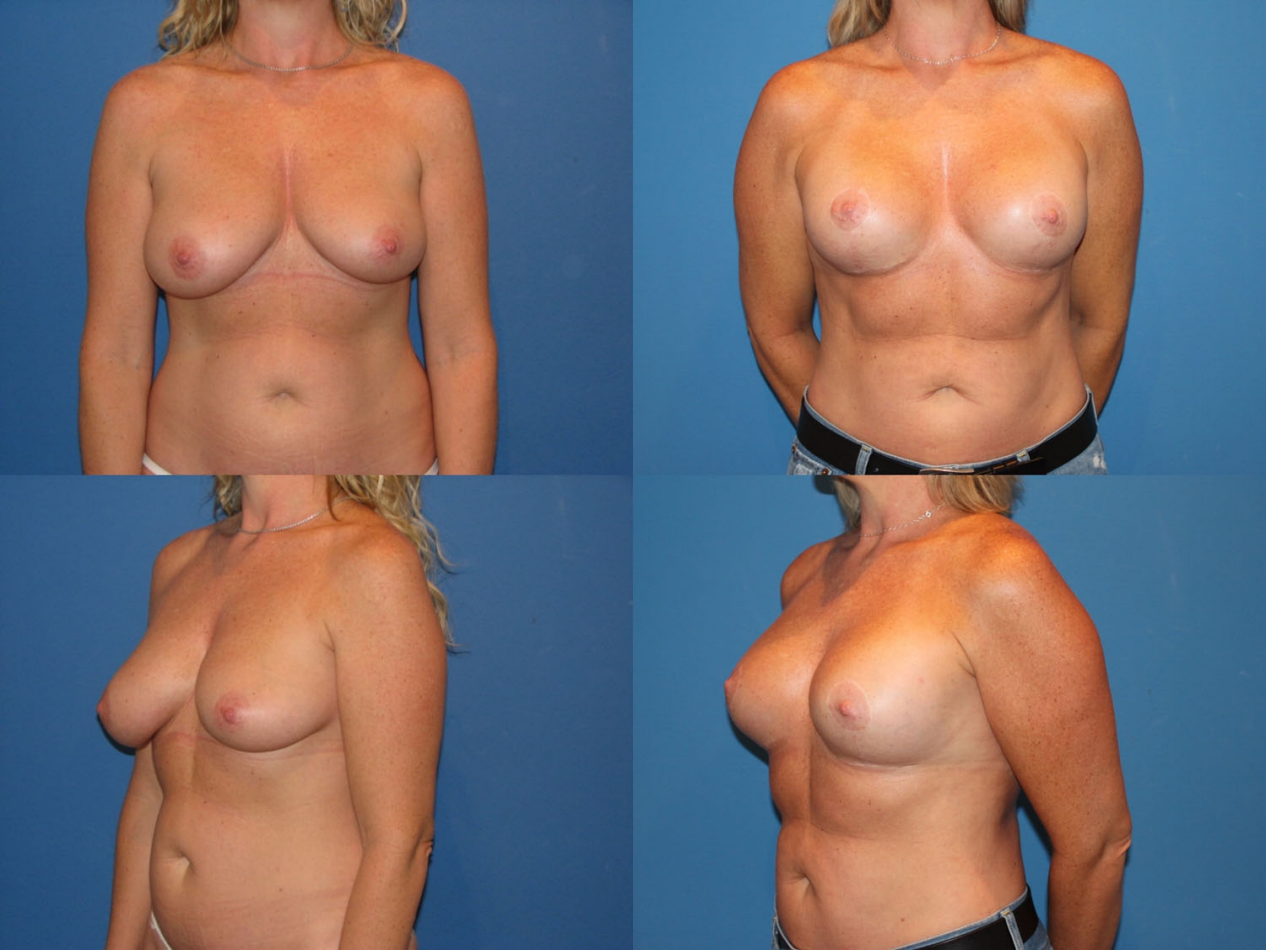
A 44-year-old female G1P1 with significant breast asymmetry who desired to be a medium to large C cup with 3/5 in definition. Reductive augmentation was performed removing 500 g from the right breast and 300 g from the left breast, and Allergan Style 45 320 cc implants were placed bilaterally. Eighteen months postoperatively

**Fig. 6:**
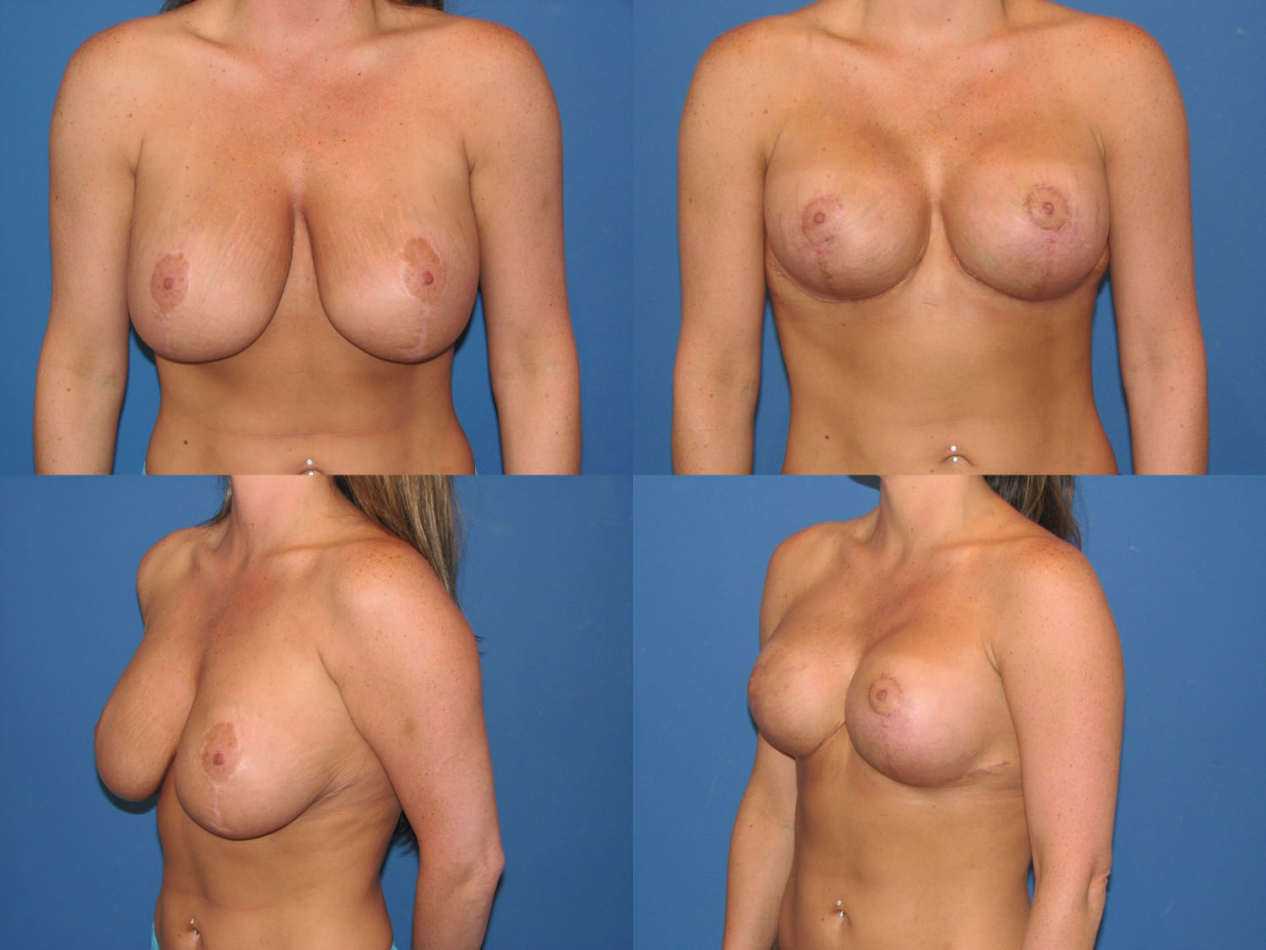
A 37-year-old female G2P2 S/P with previous breast augmentation and mastopexy who developed bottoming out and pseudoptosis. She desired to be a medium to large C cup with 3/5 in definition. Reductive augmentation was performed removing saline implant 275 cc filled to 300 cc from each breast and a tissue excision of 340 g from the right breast and 320 g from the left breast. Allergan Style 45 320 cc implants were placed bilaterally. Three months postoperatively

**Fig. 7:**
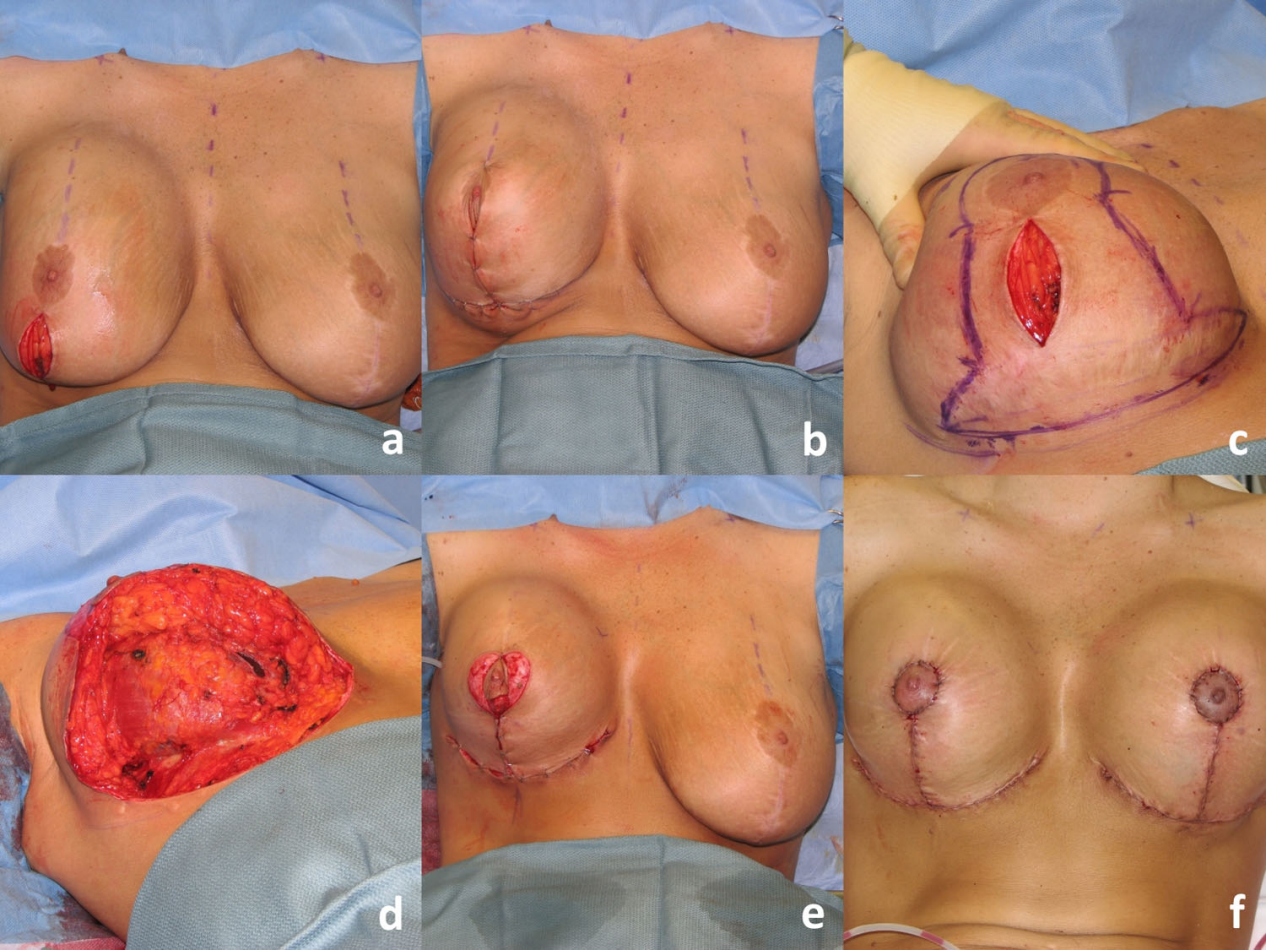
Operative sequence of patient in Fig. 6: **a** Infra-areolar vertical approach, lateral and inferior capsulorrhaphy, mirror-image capsulotomy, and placement of sizer, **b** tailor-tack mastopexy in sitting position, **c**, **d** tissue excision, **e** temporary closure, **f** final on-table result in sitting position

**Fig. 8:**
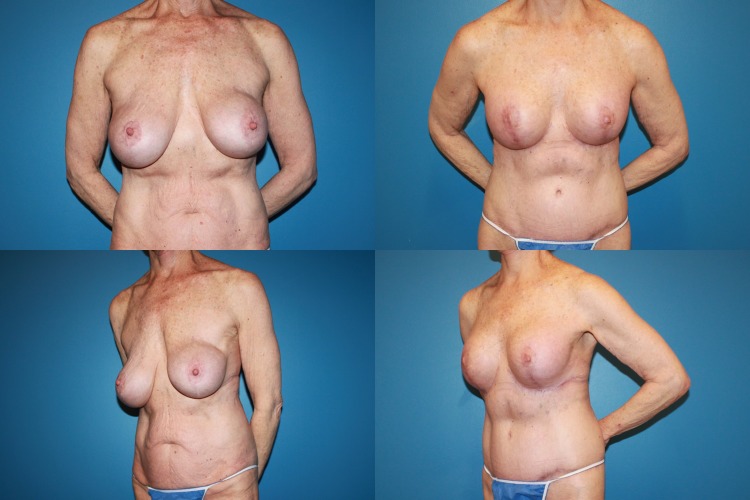
A 70-year-old female G2P2 S/P with subglandular breast augmentation and mastopexy 30 years prior with significant “slide down” of her breasts (sternal notch-to-IMC distance of 28 cm and sternal notch-to-nipple distance of 28 cm). She desired to be a medium C cup with 2-3/5 in roundness and significant lifting of her breasts. Reductive augmentation was performed removing CUI saline breast implants 270 cc filled to 275 cc on the right and 300 cc filled to 450 cc on the left. The implants were repositioned in the subpectoral location. An Allergan SRF 325 cc implant was placed on the right and SRF 345 on the left. A total of 230 g of breast tissue was removed from the right breast and 155 g from the left breast. One year postoperatively

**Fig. 9:**
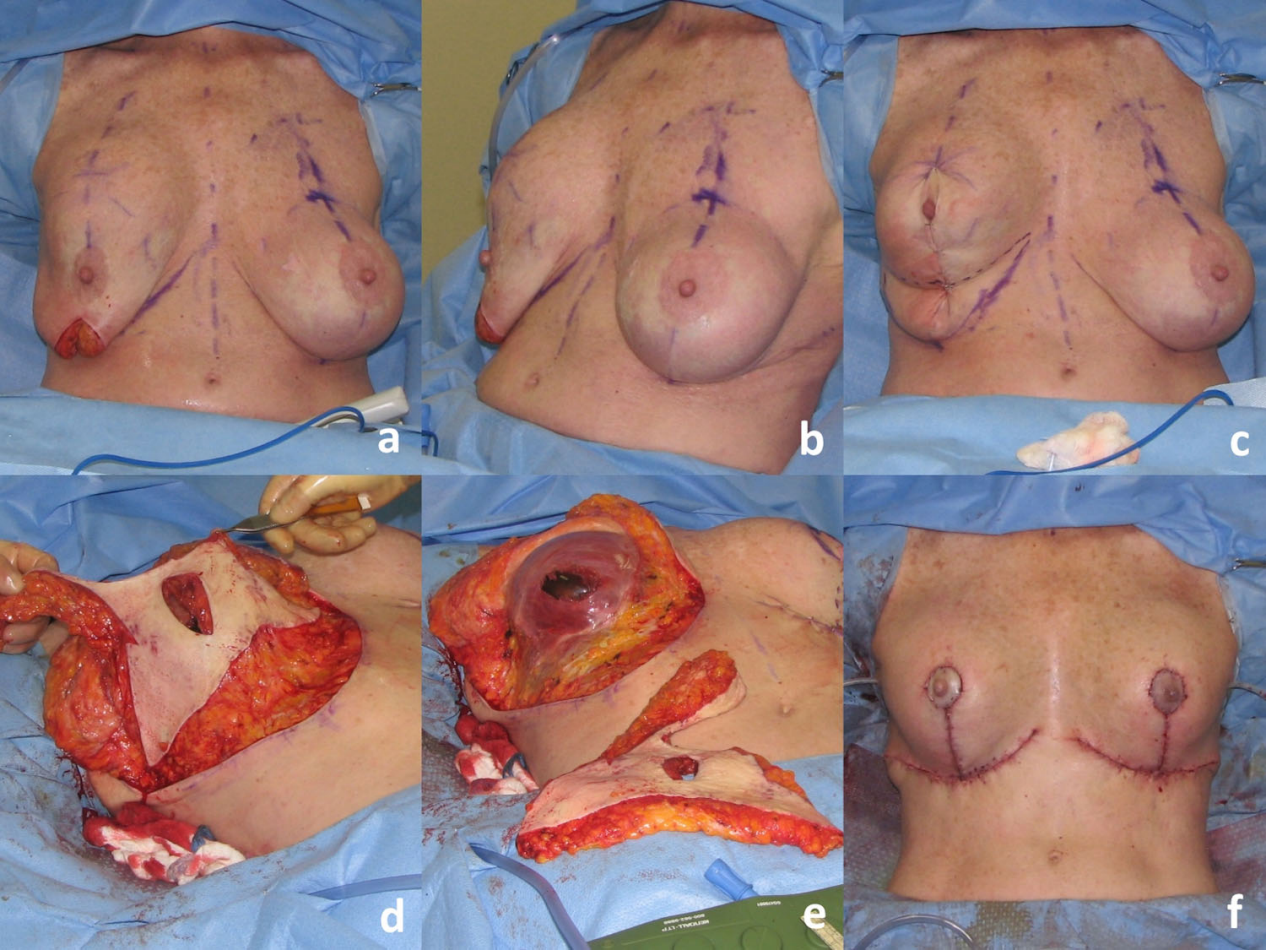
Operative sequence of patient in Fig. 8: **a**, **b** infra-areolar approach, reposition of sizer in subpectoral location. Note elevation of implant mound, **c** tailor-tack mastopexy. Note significant elevation of IMC and redundancy of inferior pole breast tissue, **d**, **e** tissue excision. The patient required undermining of the upper abdomen and elevation of the IMC prior to closure, **f** final on-table result in sitting position

**Fig. 10:**
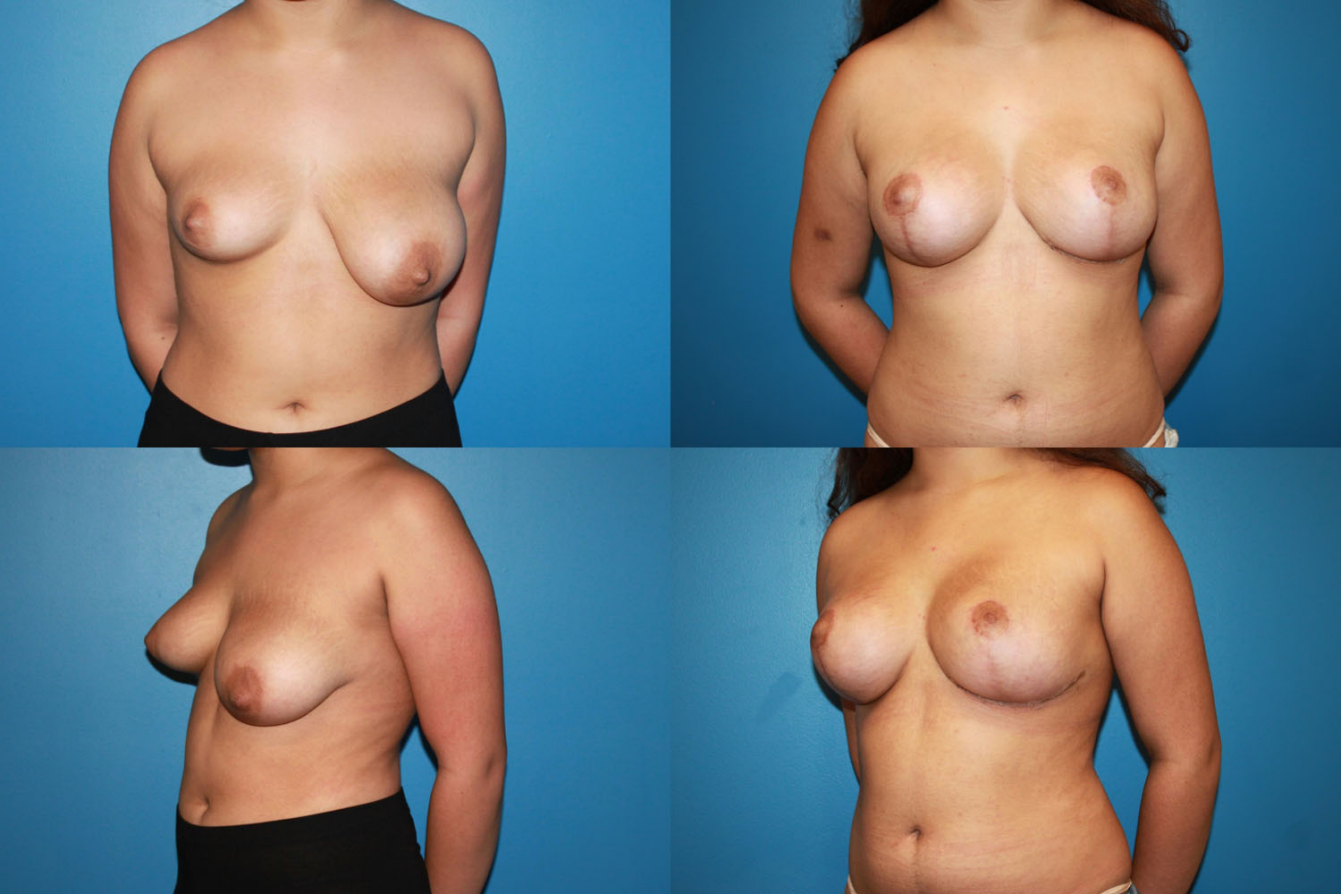
A 17-year-old female G0 with significant breast asymmetry who desired to be a large C cup with 2/5 in definition. Reductive augmentation was performed removing 55 g from the right breast and 290 g from the left breast, and Allergan SRF 385 cc implants were placed bilaterally. One year postoperatively

Between May 2013 and June 2017, 35 consecutive reductive augmentations were performed. Of these, 12 patients had primary macromastia (Figs. 3, 4, 5), while 23 patients had undergone previous augmentation (Figs. 6, 7, 8, 9). Six patients had gross breast asymmetry, defined as a tissue excision of 75 g or greater between breasts (Fig. 10). The average patient age was 45 (range 17–73) years. Patients had an average of 26 months (range 3–48 months) of follow-up. The operative time ranged from 4 to 6½ h.

For the primary group, the average tissue removal was 255 g (range 55–465 g) per breast and the average implant placed was 326 cc (range 230–470 cc) with a net increase in total volume per breast of 71 g (range + 145 to – 218 g).

In the revision group, the average tissue removal was 227 g (range 55–570 g), the average implant removed was 362 cc, and the average implant placed was 391 cc (range 230–600 cc) with a net decrease in total volume of 198 cc (range − 15 to – 380 g). It is assumed that one gram of breast tissue equals one cc. In this group, three patients had implants in the subglandular position and required a site change to a subpectoral location, three patients had a prior breast reduction (unknown pedicle), and 14 had a previous mastopexy.

For patients with gross asymmetry (over 75-g excision compared to contralateral side), there were a total of 6 patients, 3 primaries and 3 revisions (included in above results). An average of 137 g (range 75–180 g) more tissue was removed from the larger breast than the smaller breast, and all received similar-sized implants (within 30 cc) bilaterally.

Seven patients required subsequent revision, revision rate of 20%. Bottoming out occurred in a total of 6 patients, 4 were mild and required excision of the IMC under local anesthesia and 2 required major revisionary surgery under general anesthesia. Note, in both patients, there were multiple previous breast surgeries weakening the tissues of the inferior pole of the breast. Two patients desired a larger implant placed. No nipple necrosis was noted, and surprisingly, there were no capsular contractures reported.

## Discussion

Several techniques have been described to improve superior pole fullness utilizing either de-epithelialized or parenchymal flaps, or mesh [14, 15], but these have produced inconsistent long-term results at best [7, 16]. Simultaneous or staged breast augmentation with mastopexy has been described [3, 9, 17–19], but these are not associated with large reductions in the inferior pole breast tissue. Notably, the Regnault “minus-plus mastopexy” [20, 21] is a combination of augmentation and mastopexy, involving tissue excision from the lower quadrants and an inferolateral-based flap that is pulled medially to redefine the inframammary crease. Although this procedure does provide more fullness in the upper quadrants than mastopexy alone, the degree of upper pole breast fullness and inframammary crease elevation is limited due to the inferiorly based flap when compared to reductive augmentation. Another approach to achieving upper pole fullness without augmentation has been described by Biggs and Graf [16, 22]. This procedure involves mobilization of a chest wall-based flap of breast tissue passed under a loop of pectoralis major muscle with subsequent mastopexy. Because an implant is not utilized, the degree of fullness and overall roundness of the breasts are less than can be achieved with reductive augmentation. Additionally, because the flap is based inferiorly, there is again limitation in the elevation of the inframammary crease and breast.

The evolution of the reductive augmentation came on the heels of the Lejour and Hall-Findlay breast reductions [4, 6]. Once the learning curve of a superomedial-based circumvertical reduction had been overcome, it became apparent that the placement of a submuscular breast implant was conceptually easy since the pectoralis major muscle was clearly exposed during the dissection. We initially performed the reduction first, followed by the augmentation, and encountered two technical challenges similar to those previously described by Persoff [3, 18]. The first issue was an excess of medial fullness, placing the maximum projection medial to the areola. This was resolved with greater tissue removal medially and less laterally as well as further undermining of the medial and lateral flaps. The other issue was the thinness of the tissue inferomedial aspect of the partial submuscular pocket (inferior to the inferior aspect of the pectoral major muscle). This layer was easily torn by retraction or manipulation, particularly while releasing the inferomedial fibers of the pectoralis major muscle. Uncorrected, such a tear would interrupt complete coverage of the implant and result in a significant bulge. The integrity of this thin layer therefore limited the inferior dissection of the submuscular pocket, and ultimately the size of the implant that could be placed.

It was because of these difficulties that another approach was attempted. In current reductive augmentation, the implant is placed first via an infra-areolar vertical incision. There is little potential interruption of the nipple-areolar blood supply, and there is a greater degree of exposure. A standard subpectoral breast augmentation is carried out without the limiting factors of thinning of the inferomedial dissection or implant size. The subsequent excision of breast tissue is easily performed while preserving the tissue surrounding the implant inferomedially. At the time of the first tailor-tack mastopexy with the patient in the upright position, it becomes apparent that the implant appears quite high and that a greater elevation of the nipple-areolar complex is required than originally expected. A superomedial-based nipple-areolar pedicle is crucial when elevating the areola more than 4 cm which is very common in this operation. It also is safe since 14 patients had a previous mastopexy and 3 patients had a previous breast reduction without nipple-areolar necrosis. The superomedial pedicle also allows a greater excision of the inferior pole of the breast, and because there is a significant volume reduction in the inferior portion of the breast, the breast mound and inframammary crease are significantly elevated with this procedure. When making preoperative and operative markings it is important to consider that the corresponding nipple-areolar complex will need to be placed higher than in standard reductions.

Other technical issues that required refinement resulted from a significant degree of breast and inframammary crease elevation. This often leads to excessive redundant tissue in the inferior pole of the breast or large inferior dog-ear. Consequently, several early patients required revisions for conversion to an inferior crescent incision or dog-ear removal, respectively. We now perform a transverse excision at the time of surgery if there is a significant amount of redundant tissue, eliminating the need for a later revision. By measuring the areola-to-IMF distance, the length of the IMF incision can be predicted and discussed with the patient. A-IMF distances greater than 7 cm will generally need larger excisions at the IMF. Typically, those patients with a preoperative A-IMF distance of greater than 9 cm will require a full anchor incision. Additionally, measuring the sternal notch-to-IMF distance is also helpful in the prediction of IMF elevation. SN-IMF distances normally range around 21–22 cm. Those that are 23 cm or greater will most likely need larger excisions along the IMF. In our series, the lowest breast had a measurement of 28 cm.

Another technical point that was encountered was the shape of the breast changed significantly after larger tissue excisions. Once the previous mastopexy incision was re-stapled, the breast appeared flat on the bottom and the areolar opening was significantly larger. Two maneuvers that helped re-establish the attractive appearance of the breast were a generous undermining of the medial and lateral flaps, so they could advance to the midline without tethering and the other is “secondary” mastopexy. In the secondary mastopexy, the patient is placed back in a sitting position and re-stapled to achieve the aesthetic appearance of the breast and reduce the size of the areolar opening.

In the revisionary cases, especially when changing out to a smaller implant, there was noted to be some redundancy of the capsule inferiorly even after aggressive capsulorrhaphy. This was addressed either with excision of a portion of the capsule or with “external” capsulorrhaphy sutures to add to the superior elevation of the implant. A key point is the reduction in tension on the skin on the inferior pole of the breast. This is accomplished by elevation of the implant using the periprosthetic tissues and suturing of the medial and lateral pillars.

Reductive augmentation can be especially useful in patients with significant asymmetry in size and/or ptosis. Conventional methods of matching breast size in initially asymmetric patients, such as performing unilateral augmentation with contralateral mastopexy, or placing implants of disparate volume, can result in breasts that differ markedly in shape. In reductive augmentation, the size and shape of the breasts are better matched by removing the disparity in breast volume and placing implants of the same or similar size [23].

The different patterns of volume change between patients undergoing primary breast surgery and those previously augmented warrant further discussion. For individuals who had not been previously augmented, the average amount of tissue removed per breast was less than the average volume of the implant placed by 71 g. Although this amounts to a modest increase in net volume, breasts appear much smaller postoperatively because the volume is redistributed in a higher breast with more projection, less width, and greater superior pole fullness. Patients who had been augmented previously experienced more significant decreases in net breast volume, an average of 198 g. In many instances, the patient’s implants were replaced by a higher-profile implant to achieve a round result. Many patients wanted to be smaller, but some wanted to be larger; however, they all wanted removal of inferior pole breast tissue and more upper pole fullness.

A 20% revision rate might seem high for most operations; however, only three (8.6%) required general anesthesia, and the rest were small revisions under local anesthesia. Recent publications have a revision rate of 14.6 and 23.2% for one-stage breast augmentation and mastopexy [24, 25]. Reductive augmentation is a huge undertaking with many steps and a three-dimensional remolding of the breast. It is important to sit the patient up after each major manipulation to achieve symmetry at each step and not get lost in the surgery. It is also crucially important to relay this to the patient, so they can accept and/or expect a minor or major revision, especially when starting to do this type of surgery. All of the techniques are standard surgical procedures that experienced breast surgeons perform; however, linking them into one operation can be challenging.

A discussion involving the pros and cons of performing a one-stage surgery versus a two-stage surgery in which a breast reduction is performed first and then a breast augmentation later can be debated. Reductive augmentation as presented allows a one-stage surgery to achieve a specific result in those patients who desire a round-appearing breast with greater upper pole fullness.

## Conclusion

Reductive augmentation is a technique for creating a round breast with more upper pole fullness in those patients who are otherwise candidates for breast reduction and desire a specific aesthetic appearance. This procedure is especially helpful in those patients with asymmetry and/or patients with previous breast augmentation with or without mastopexy who develop pseudoptosis and want a more round-appearing breast. The procedure can result in an increase or decrease in overall volume and redistribute the remaining volume into a higher position. Even in those patients who experience minimal reduction in net breast weight, the breast appears to be smaller due both to rounder shape, reduction in the skin envelope, and to redistribution of volume. We have described a technique for a certain subsegment of patients who have larger breasts, ptosis, and who desire a more round-appearing result with upper pole fullness.
